# Identification, isolation, and expression analysis of heat shock transcription factors in the diploid woodland strawberry *Fragaria vesca*

**DOI:** 10.3389/fpls.2015.00736

**Published:** 2015-09-15

**Authors:** Yang Hu, Yong-Tao Han, Wei Wei, Ya-Juan Li, Kai Zhang, Yu-Rong Gao, Feng-Li Zhao, Jia-Yue Feng

**Affiliations:** ^1^State Key Laboratory of Crop Stress Biology for Arid Areas, College of Horticulture, Northwest A&F UniversityYangling, Shaanxi, China; ^2^Key Laboratory of Protected Horticulture Engineering in Northwest China, Ministry of AgricultureYangling, Shaanxi, China

**Keywords:** strawberry (*Fragaria vesca*), heat shock transcription factor, expression analysis, subcellular localization, heat stress, abiotic stress, phytohormones

## Abstract

Heat shock transcription factors (Hsfs) are known to play dominant roles in plant responses to heat, as well as other abiotic or biotic stress stimuli. While the strawberry is an economically important fruit plant, little is known about the Hsf family in the strawberry. To explore the functions of strawberry Hsfs in abiotic and biotic stress responses, this study identified 17 *Hsf* genes (*FvHsfs*) in a wild diploid woodland strawberry (*Fragaria vesca*, 2*n* = 2*x* = 14) and isolated 14 of these genes. Phylogenetic analysis divided the strawberry *FvHsfs* genes into three main groups. The evolutionary and structural analyses revealed that the *FvHsf* family is conserved. The promoter sequences of the *FvHsf* genes contain upstream regulatory elements corresponding to different stress stimuli. In addition, 14 FvHsf-GFP fusion proteins showed differential subcellular localization in *Arabidopsis* mesophyll protoplasts. Furthermore, we examined the expression of the 17 *FvHsf* genes in wild diploid woodland strawberries under various conditions, including abiotic stresses (heat, cold, drought, and salt), biotic stress (powdery mildew infection), and hormone treatments (abscisic acid, ethephon, methyl jasmonate, and salicylic acid). Fifteen of the seventeen *FvHsf* genes exhibited distinct changes on the transcriptional level during heat treatment. Of these 15 *FvHsfs*, 8 *FvHsfs* also exhibited distinct responses to other stimuli on the transcriptional level, indicating versatile roles in the response to abiotic and biotic stresses. Taken together, the present work may provide the basis for further studies to dissect *FvHsf* function in response to stress stimuli.

## Introduction

Global warming has brought about many severe environmental problems with adverse impacts on almost all aspects of plant development, growth, reproduction, or yield (Hedhly et al., 2009; Mittler et al., 2012). Although plants are sessile organisms that cannot escape from stress conditions, a cascade of activities regulated by transcription factors are triggered in response to such conditions (Schwechheimer and Bevan, 1998). Particularly, plant heat shock transcription factors (Hsfs) are known to play a central role in protecting plants from heat or other stress conditions (Scharf et al., 2012).

Hsfs are structurally conserved throughout the eukaryotic kingdom. Earlier studies focusing on the structural and biochemical characteristics of Hsfs (Harrison et al., 1994; Littlefield and Nelson, 1999) revealed that a typical Hsf consists of five conserved motifs, including a DNA-binding domain (DBD), an oligomerization domain (OD), a nuclear localization signal (NLS), a nuclear export signal (NES), and an activator peptide motif (AHA) (Never et al., 1996; Scharf et al., 2012).

A number of studies have demonstrated that Hsfs play vital roles in plant responses to abiotic stresses such as heat, cold, salt, drought, and oxidative conditions, among others (Schramm et al., 2008; Yoshida et al., 2010; Hwang et al., 2014). For example, a versatile regulatory regime involving HsfA1, HsfA2, HsfB1a, Hsp90, and Hsp70 in the control of heat stress response was proposed (Hahn et al., 2011). HsfA2 serves as the most strongly induced Hsf, accumulating to high levels after exposure of tomato (Chan-Schaminet et al., 2009), *Arabidopsis* (Schramm et al., 2006), or rice (Mittal et al., 2009) to long-term heat stress. Substantial evidence has also demonstrated that HsfA2 is associated with the expression of multiple Hsps or general stress-related non-chaperone encoding genes (Schramm et al., 2006; Chan-Schaminet et al., 2009; Nishizawa-Yokoi et al., 2009). HsfA3 is located downstream of the DREB2A stress-regulatory system in the transcriptional cascade (Schramm et al., 2008) and could be involved in physiological responses to drought and salt stress (Li et al., 2013). HsfA4 was reported to be involved in oxidative stress (Davletova et al., 2005) and cadmium tolerance in rice and wheat (Shim et al., 2009). HsfA6a and HsfA6b expression is highly increased under high salinity or dehydration conditions (Yoshida et al., 2010; Hwang et al., 2014). In addition, HsfB1a and HsfB2a/b were reported to be associated with pathogen resistance in *Arabidopsis* (Kumar et al., 2009; Ikeda et al., 2011; Pick et al., 2012). In addition to stress resistance, Hsfs have also been linked to important roles in plant growth and development (Kotak et al., 2007; Almoguera et al., 2009). Recently, genome-wide expression profiles analyses in rice (Mittal et al., 2009), wheat (Xue et al., 2014), soybean (Chung et al., 2013), and apple (Giorno et al., 2012) also indicated that several *Hsf* genes were transcribed at high levels during heat, cold, salt, and drought stresses. However, little is known about *Hsf* genes in the strawberry.

The strawberry is one of the most important fruit crops in the world. However, more extreme temperatures and droughts, serious fungal infections and secondary salinization have strongly limited the growth, development, reproduction, and yield of strawberry plants in recent years (Maughan et al., 2015; Nezhadahmadi et al., 2015). The woodland strawberry *F. vesca* has a smaller genome (~240 Mb) that is highly congenic with its octoploid cultivated species (*Fragaria* × *ananassa* Duch.) (Shulaev et al., 2011). These features underlie its potential utility as a versatile experimental system for studying the counterparts of many important genes in Rosaceae fruit crops (Shulaev et al., 2011; Liu et al., 2014).

To explore the functions of Hsfs in abiotic and biotic stress responses in the strawberry, the current study identified and isolated *FvHsf* genes in a diploid woodland strawberry accession Heilongjiang-3 (Liu et al., 2014), in addition to analyzing the evolutionary relationships, gene structure, protein domains, and expression profiles of these genes with respect to heat, drought, salt, cold, and powdery mildew infection stresses and abscisic acid, ethephon, methyl jasmonate, and salicylic acid treatments. Notably, the subcellular localization of most (14/17) of the FvHsf proteins was demonstrated; these data have not been fully elucidated in any other plant species. Taken together, the present work may provide the basis for further studies to dissect *FvHsf* function in response to abiotic or biotic stresses.

## Materials and methods

### Identification and classification of *Hsf* genes in strawberry

A publicly available database (http://planttfdb.cbi.pku.edu.cn/) predicted that 15 *Hsf* genes exist in the diploid strawberry genome (accession Hawaii-4) and predicted the corresponding nucleotide and amino acid sequences. All of the nucleotide and amino acid sequences of the predicted *FvHsf* genes were used as queries to perform BLAST searches in the public NCBI database. The strawberry genes with the highest identity (>90%) and lowest *E*-value (0) were selected as candidates. If two or more protein sequences at the same gene locus overlapped, only the longest sequence was used. All of the putative candidates were manually verified with the InterProScan program (http://www.ebi.ac.uk/Tools/pfa/iprscan/) to confirm their completeness and the presence of a DNA-binding domain (PF00447) (http://pfam.sanger.ac.uk/).

Phylogenetic analysis was used to classify the *FvHsf* genes into three classes and further subclasses (Scharf et al., 2012). The full-length amino acid sequences of Hsf proteins from strawberry (*FvHsf*), *A. thaliana* (*AtHsf*), rice (*Oryza sativa* L., *OsHsf*) (Guo et al., 2008), apple (*Malus domestica* Borkh, *MdHsf*) (Giorno et al., 2012), and grape (*Vitis vinifera, VvHsf*) were used to generate a phylogenetic tree through ClustalW alignment and the unrooted Neighbor-joining method using MEGA 5.0 (Tamura et al., 2011). Neighbor-joining analysis with pairwise deletion was performed using the Jones-Taylor-Thornton (JTT) model. Bootstrap analysis was performed with 1000 replicates to assess the level of statistical support for each tree node (Xue et al., 2014).

### Gene structure, protein domains, and synteny analysis of strawberry *Hsf* genes

The exon-intron structures of *FvHsf* genes were visualized using the online program GSDS 2.0 (http://gsds.cbi.pku.edu.cn/), which aligns the respective coding sequences with corresponding full-length sequences. The protein domains of the *FvHsf* genes were identified using MEME online tools (http://meme.nbcr.net/meme/) with the parameters set as follows: the minimum length of the conserved motif was 6, the maximum length of the conserved motif was 100, and the largest number of discovered and conserved motifs was 25. The other parameters retained their default settings. The results were presented by DOG 1.0 (Ren et al., 2009). The syntenic blocks used to construct a synteny analysis map between the strawberry and *Arabidopsis Hsf* genes were obtained from the Plant Transcription Factor Database (http://planttfdb.cbi.pku.edu.cn/) and Plant Genome Duplication Database (Lee et al., 2013), and the diagrams were generated by the Circos program, version 0.63 (http://circos.ca/).

### Isolation and subcellular localization of strawberry *Hsf* genes

To provide more insight into the function of *FvHsfs*, we designed gene-specific primers to isolate the putative *Hsf* genes from a diploid woodland strawberry accession Heilongjiang-3. The predicted full-length coding sequences of *FvHsf* genes were amplified from Heilongjiang-3 cDNA using high-fidelity Taq HS-mediated PCR. Then, we used the isolated *FvHsfs* to examine the subcellular localization of these proteins. The amplified PCR products were digested with *Xba*I/*Xho*I and *Kpn*I and fused in-frame with GFP in the *Xba*I/*Xho*I and *Kpn*I site of the pBI221 vector containing the CaMV *35S* promoter (Clontech, Beijing, China), resulting in the pFvHsf-GFP plasmids. The primers used to clone genes and construct vectors are available in Supplementary Table S1.

For the transient expression of FvHsfs-GFP in *Arabidopsis* mesophyll protoplasts, the corresponding pFvHsfs-GFP plasmid was transformed into *Arabidopsis* mesophyll protoplasts using the PEG-calcium transfection method described in Yoo et al. (2007). After transformation, the *Arabidopsis* mesophyll protoplasts were kept in darkness at room temperature for 16–18 h before examination by fluorescence microscopy. Images were acquired using an Olympus BX-51 inverted fluorescence microscope (Olympus, Japan). The image data were processed using Adobe Photoshop (Mountain View, CA, USA). All of the transient expression assays were repeated at least three times.

### *In silico* promoter analysis

The *cis*-elements presented in the 1 kb region upstream of the translation start site of the strawberry *Hsf* genes were obtained from the PlantCARE database (Rombauts et al., 1999), and the corresponding nucleotide sequences were retrieved from NCBI with their gene IDs (http://www.ncbi.nlm.nih.gov/).

### Plant materials and treatments

The wild diploid strawberry *F. vesca* accession Heilongjiang-3 was grown in the strawberry germplasm resource greenhouse of Northwest A&F University. The potted strawberry plants were grown at 22°C with 70% relative humidity and no supplemental light. Six-month-old strawberry seedlings with the tenth leaf fully expanded were selected for treatments. The roots, stems, leaves, flowers, receptacles, and fruits of greenhouse-grown Heilongjiang-3 were used to analyze the organ-specific expression of the *FvHsf* genes. *A. thaliana* ecotype Col-0 was grown at 22°C with 75% relative humidity under short-day (8 h light at 125 μmol ·m^−2^·s ^−1^, 16 h dark) conditions for 4–5 weeks before transformation.

Heat stress treatment was performed by transferring potted strawberry plants to a 42°C chamber for 48 h. Leaves were collected from the plants at 0, 0.5, 2, 4, 8, 12, 24, and 48 h post-treatment (hpt). Cold stress treatment was performed by transferring the plants to a 4°C chamber for 48 h. Leaves were harvested from the plants at 0, 0.5, 2, 4, 8, 12, 24, and 48 hpt. For both the heat and cold treatments, another set of potted Heilongjiang-3 seedlings were kept in the control temperature range of 22–27°C. Salt stress was simulated by irrigating potted strawberry plants with 300 mM NaCl once. Another set of control Heilongjiang-3 seedlings was similarly treated with distilled water. The leaves were collected from the plants at 0, 0.5, 2, 4, 8, 12, 24, and 48 hpt. Drought stress was simulated by withholding water and sampling at 0, 24, 48, 72, 96, 120, and 144 hpt. The plants were rewatered after 144 h of drought stress and sampled again 24 h later. The plants were inoculated with powdery mildew (*Podosphaera aphanis*) by touching the adaxial epidermis of Heilongjiang-3 with sporulating colonies located on the leaf surface of the strawberry cv. Red cheeks. Another set of control Heilongjiang-3 seedlings was similarly touched with uninfected, healthy leaves. The treated plants were then incubated in a controlled environment. Inoculated leaves were collected at 0, 24, 48, 72, 96, 120, 144, and 168 h post-inoculation (hpi). The inoculations were repeated three times. For hormone treatments, the strawberry leaves were sprayed with a solution containing 0.1 mM abscisic acid (ABA), 1 mM salicylic acid (SA), 0.1 mM methyl jasmonate (MeJA), or 0.5 g/L ethephon (Eth), while another set of control Heilongjiang-3 seedlings were similarly sprayed with distilled water. The treated leaves were then collected at 0, 0.5, 2, 4, 8, 12, 24, and 48 hpt for RNA isolation. Six leaves from six separate plants were collected at each time point of each treatment and combined to form one sample, and all of the experiments were performed independently in triplicate.

### Semi-quantitative PCR and real-time quantitative PCR analysis

Total RNA was isolated from treated leaves or tissue samples using an EZNA Plant RNA Kit (R6827-01, Omega Bio-tek, USA). cDNA synthesis was performed using PrimeScript RTase (TaKaRa Biotechnology, Dalian, China). The strawberry *18S* gene was used as a control to normalize the amount of cDNA used from each sample (Raab et al., 2006). VECTOR NTI was used to design gene-specific primers for each *FvHsf* gene (Supplementary Table S1). The following semi-quantitative reverse-transcription PCR program was used: 95°C for 3 min, 30–32 cycles of 95°C for 30 s, 60°C for 30 s and 72°C for 30 s, and a final step of 72°C for 5 min. The PCR products were separated on a 1.0% (w/v) agarose gel, stained with ethidium bromide, and imaged under UV light for further gene expression analysis. Each reaction was performed in triplicate, with the three independent analyses for each treatment showing the same trends for each gene. The expression profiles from the semi-quantitative RT-PCR were collated, analyzed, and visualized using the GeneSnap and MeV 4.8.1 programs.

Real-time quantitative PCR was carried out using SYBR green (TaKaRa Biotechnology) on an IQ5 real time-PCR machine (Bio-Rad, Hercules, CA, USA) with a final volume of 21 μl per reaction. Each reaction mixture contained 10.5 μl SYBR Premix Ex Taq II (TaKaRa Biotechnology), 1.0 μl cDNA template, 1.0 μl of each primer (1.0 μM), and 7 μl sterile distilled H_2_O. Each reaction was performed in triplicate. The cycling parameters were 95°C for 30 s, 40 cycles at 95°C for 30 s and 58°C for 30 s. After amplification, the samples were kept at 50°C for 1 min and the temperature was gradually raised by 0.5°C every 10 s to perform the melt-curve analysis. Relative gene expression was determined using an 18S-26S interspacer gene as an endogenous control gene (Raab et al., 2006). Each relative expression level was analyzed with IQ5 software using the Normalized Expression method. The primers used for real-time RT-qPCR are listed in Supplementary Table S1.

### Statistical analysis

The data are presented as the mean ± standard deviation of the mean (SD) and were tested for statistical significance with a paired Student's *t*-test (http://www.physics.csbsju.edu/stats/). *p* < 0.05 was selected as the point of minimal statistical significance in all of the analyses.

## Results

### Identification and isolation of *Hsf* genes in the diploid strawberry

A total of 17 strawberry *Hsf* genes were originally obtained through the BLAST search in NCBI using the 15 strawberry Hsf amino acid sequences that were predicted in the sequenced genome of the accession Hawaii-4 (http://planttfdb.cbi.pku.edu.cn/) (Shulaev et al., 2011). All 17 of the proteins contained apparently complete Hsf-type DNA-binding domains (PF00447) and oligomerization domains (Never et al., 1996; Scharf et al., 2012). As a result, 17 FvHsf members were identified in the diploid strawberry (Table 1). More detailed information about each *FvHsf* gene, including the *Hsf* gene IDs, gene location, length of the coding sequences, and the characteristics of the FvHsf proteins can be found in Table 1. Using the predicted *FvHsf* coding sequences, we initially isolated 14 homologous genes from a diploid woodland strawberry accession Heilongjiang-3 (*F. vesca*). It is notable that the ORF sequences of the isolated *FvHsfs* from accession Heilongjiang-3 share high identities (≥96%, *E*-value = 0) with the corresponding *FvHsfs* in strawberry accession Hawaii-4 (Shulaev et al., 2011) (Table 1).

**Table 1:** **Characteristics of strawberry ***Hsf*** genes**.

**Name**	**Gene ID^a^**	**Gene location**	**Length (bp)**	**Number of aa**	**MW^b^ (kDa)**	**Isoelectric point^b^**	**Identity/ *E*-value^c^**	**Accession numbers^d^**
*FvHsfA1b*	101302336	LG3: 11614311–11618187	3876	494	55.01	5.02	99%, 0.0	KT283219
*FvHsfA1d*	101302657	LG5:21020179–21025194	5015	508	55.66	4.80	99%, 0.0	KT283220
*FvHsfA2a*	101312795	LG2:20570433–20572945	2512	372	41.97	4.86	99%, 0.0	KT283221
*FvHsfA3a*	101295258	LG6:13129046–13132026	2980	456	50.35	4.77	99%, 0.0	KT283222
*FvHsfA4a*	101311435	LGun:147013–149497	2484	497	55.72	5.13	99%, 0.0	KT283223
*FvHsfA4b*	101290970	LG6:16216378–16219992	3614	439	49.77	4.99	–	–
*FvHsfA5a*	101302160	LG4:14062542–14066187	3645	578	64.93	7.40	99%, 0.0	KT283224
*FvHsfA6a*	101307359	LG2:16115738–16118875	3137	362	41.18	5.79	99%, 0.0	KT283225
*FvHsfA7a*	101297339	LG4:5513754–5515879	2125	345	40.13	5.42	–	–
*FvHsfA8a*	101290755	LG4:19035204–19038162	2958	474	54.45	4.99	–	–
*FvHsfA9a*	101302973	LG1:6313891–6316458	2567	447	50.90	5.23	99%, 0.0	KT283226
*FvHsfB1a*	101294882	LG1:10956423–10958906	2483	290	32.01	5.96	99%, 0.0	KT283227
*FvHsfB2a*	101299242	LG7:22683186–22684501	1315	289	31.97	8.82	98%, 0.0	KT283228
*FvHsfB2b*	101301205	LG5:1089695–1092781	3086	355	38.41	4.79	96%, 0.0	KT283229
*FvHsfB3a*	101307517	LG6:33122976–33124719	1743	236	27.10	5.91	99%, 0.0	KT283230
*FvHsfB4a*	101301353	LG2:21014849–21016923	2074	386	43.21	7.73	99%, 0.0	KT283231
*FvHsfC1a*	101304412	LG1:3848442–3850066	1624	362	40.49	5.77	99%, 0.0	KT283232

a *IDs are available in the strawberry Genome Database (http://www.rosaceae.org)*.

b *Detailed FvHsf protein characteristics were predicted by ExPASy online service (http://web.expasy.org/cgi-bin/compute_pi/pi_tool)*.

c *The identities of the isolated FvHsf genes were derived from a NCBI Blast search using the FvHsf Heilongjiang-3 nucleotide sequences*.

d *Sequence data of isolated FvHsfs can be found in GenBank databases using these accession numbers*.

### Phylogenetic analysis and classification of FvHsf proteins

**Figure 1:**
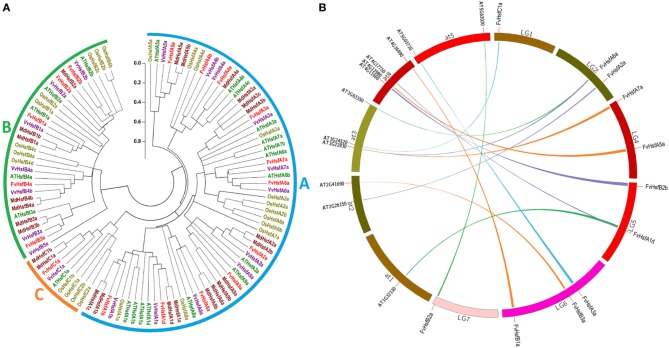
**Comparative analysis of strawberry ***Hsf*** genes with the corresponding genes in rice, apple, grapevine, and ***Arabidopsis***. (A)** The linearized neighbor-joining phylogenetic tree of Hsf proteins from strawberry, rice, apple, grapevine, and *Arabidopsis*. The full-length amino acid sequences of the Hsf proteins were used to construct the phylogenetic tree using the MEGA 5.0 program. Unrooted neighbor-joining analysis was performed with pairwise deletion and the Jones-Taylor-Thornton (JTT) model. The filled circle lines were used to cluster the genes into the A, B, and C classes. **(B)** Synteny analysis of strawberry and *Arabidopsis Hsf* genes. The strawberry and *Arabidopsis* chromosomes are depicted as a circle. The approximate location of each *AtHsf* and *FvHsf* gene is marked with a short red line on the circle. The colored curves denote the syntenic regions of the strawberry and *Arabidopsis Hsf* genes.

Compared with fungi and animals, plants possess large numbers of *Hsf* genes (Von Koskull-Doring et al., 2007), and the plant *Hsf* genes were assigned into A, B, and C classes based on the diversity of the oligomerization domain (Never et al., 1996). To examine the classification of FvHsf members and to gain some insight into the potential function of FvHsf proteins from well-studied Hsfs in other plant species, we used full-length amino acid sequences to perform a phylogenetic analysis of Hsf proteins from strawberry, rice, apple, grapevine, and *Arabidopsis* (Figure 1A). The phylogenetic analysis indicated that the 106 Hsf proteins from these plants were clearly grouped into three different clades corresponding to the Hsf classes A, B, and C (Figure 1A, Table 1, and Supplementary Table S2). Within each Hsf protein class, particular clusters of orthologous and paralogous genes have been identified, showing ancestral speciation or duplication events. For example, the monocot rice has an additional C2 clade but has lost the A9 and B3 clades (Figure 1A). Although all of the clades resolved in dicots (apple, grapevine, and *Arabidopsis*) are represented in the strawberry Hsf proteins, fewer paralogous pairs may limit the size of the FvHsf family (Figure 1A).

### Syntenic relations, exon-intron organization, and protein domains of the *FvHsf* genes

**Figure 2:**
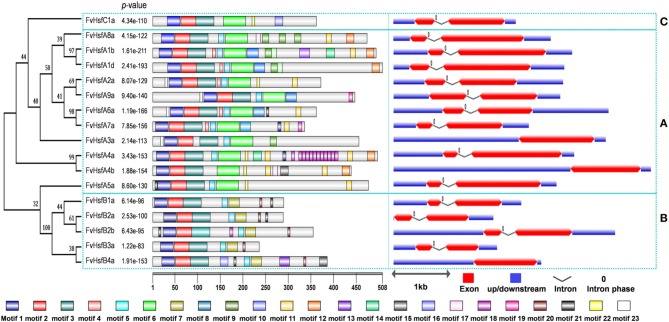
**Structural analysis of strawberry ***Hsf*** genes**. The protein domains of the strawberry *Hsf* genes are shown on the left and are denoted by rectangles with different colors. The exon-intron organization is shown on the right, with exons and introns represented by red wedges and black lines, respectively, and untranslated regions (UTRs) indicated by blue boxes. The number 0 represents the intron phase. The exon size can be estimated using the scale at the bottom of the diagram, while all of the introns were set to same length. The blue dashed rectangles are used to cluster the genes into the A, B, and C classes.

Most of the *Arabidopsis Hsf* genes have been systematically investigated in recent years, and a synteny analysis of strawberry *Hsfs* and *Arabidopsis Hsfs* was performed in the present work to ascertain whether this information might provide more functional insight. A total of 11 pairs of syntenic *Hsf* genes were found between strawberry and *Arabidopsis*; this number included 11 *FvHsf* genes and 12 *AtHsf* genes (Supplementary Table S3; Figure 1B). Interestingly, two *FvHsf* genes (*FvHsfA1d, FvHsfA6a*) and one *AtHsf* gene (*AtHsfA6b*) were found to be associated with two syntenic blocks (Supplementary Table S3). Insights into the structure of the *FvHsf* genes were obtained through an analysis of the exon/intron boundaries, which are known to play key roles in gene family evolution (Schwartz et al., 2009). As shown in Figure 2, the *FvHsf* genes exhibit a highly conserved exon-intron organization: of the 17 *FvHsf* genes, 14 possess two exons and three have one exon. It is interesting that the introns of the *FvHsf* genes divide the highly conserved DNA-binding domains coding regions into two parts, and all of the introns are phase zero introns. Plant Hsf proteins show a highly modular assembly of protein domains (Never et al., 1996; Scharf et al., 2012). We used the online MEME tool to predict the conserved FvHsf protein domains, identifying 23 conserved motifs (Figure 2; Supplementary Table S2). Unsurprisingly, all 17 of the FvHsfs showed the presence of a DNA-binding domain (DBD) in the N-terminal of the protein (Figure 2; Supplementary Table S2). The oligomerization domains (OD or HR-A/B region), which differ in sequence among the class A, B, and C members, are adjacent to the DBD (Figure 2; Supplementary Table S2). In addition, nuclear localization signal (NLS) motifs were found in 15 FvHsfs, nuclear export signal (NES) motifs were found in five class A FvHsfs, and transcription activator (AHA) motifs were found in eight class A FvHsfs (Figure 2; Supplementary Table S2). Overall, each FvHsf protein contained the necessary DBD and OD, but some FvHsfs have specific motifs such as NLS, NES, and/or AHA, which might lay the foundation for the functional divergences between different FvHsfs (Scharf et al., 1998; Döring et al., 2000; Heerklotz et al., 2001).

### Regulatory elements in the *FvHsf* promoters

**Figure 3:**
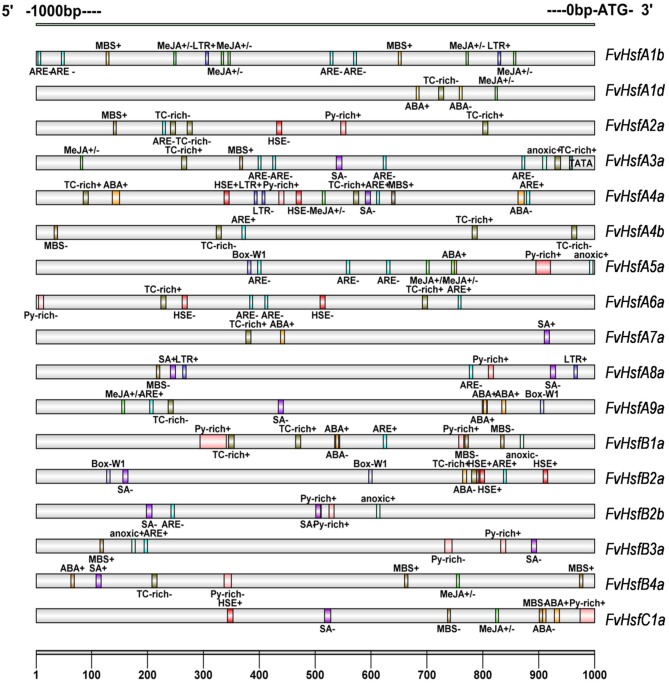
*****Cis***-elements are present in the 1 kb region upstream of the strawberry ***Hsf*** translation start site**. The analysis was performed using the PlantCARE database. The different-colored boxes mark the relative positions of the different elements.

An *in silico* survey of the putative *cis*-elements in the 1 kb region upstream of the translation initiation codon of various *FvHsf* genes showed the presence of some abiotic stress response elements such as HSEs (heat shock elements), which were found in *FvHsfA2a, FvHsfA4a, FvHsfA6a, FvHsfB2a*, and *FvHsfC1a* (Figure 3). In addition, LTR, a *cis*-acting element involved in low-temperature responsiveness (Jiang et al., 1996) was observed in *FvHsfA1b, FvHsfA4a*, and *FvHsfA8a*; anoxia responsive elements (ARE) were observed in 13 *FvHsf* genes; MBS (MYB binding site), a *cis*-acting element involved in drought-inducibility (Deng et al., 1996), was found in 10 *FvHsf* genes; Box-W1, a fungal elicitor responsive element, was identified in *FvHsfA5a, FvHsfA9a*, and *FvHsfB2a*; and TC-rich repeats, a *cis*-acting element involved in defense and stress responsiveness, was seen in 11 *FvHsf* genes. Lastly, ABA responsive elements (ABREs) were found in nine *FvHsf* genes, MeJA responsive elements (CGTCA-motif/TGACG-motif) were observed in eight *FvHsf* genes, and SA responsive elements (TCA-element) were seen in nine *FvHsf* genes (Figure 3). Notably, plenty of hormone-responsive elements were seen in the *FvHsf* promoter sequences, indicating that phytohormones could play central roles in regulation of heat stress responses (Clarke et al., 2009).

### Subcellular localization of FvHsfs

**Figure 4:**
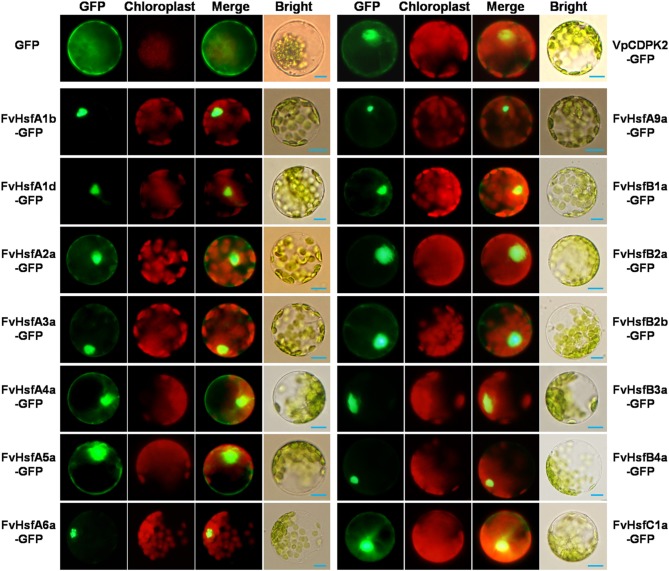
**The subcellular localization of 14 FvHsfs**. The selected *Hsf* genes were cloned from a diploid woodland strawberry (*F. vesca*) and used to construct CaMV35S::Hsfs-GFP vectors in which GFP was fused at the C terminus. The 14 FvHsf-GFP fusion proteins (FvHsfA1b-GFP, FvHsfA1d-GFP, FvHsfA2a-GFP, FvHsfA3a-GFP, FvHsfA4a-GFP, FvHsfA5a-GFP, FvHsfA6a-GFP, FvHsfA9a-GFP, FvHsfB1a-GFP, FvHsfB2a-GFP, FvHsfB2b-GFP, FvHsfB3a-GFP, FvHsfB4a-GFP, and FvHsfC1a-GFP), the VpCDPK2-GFP marker protein, and GFP control were transiently expressed in *A. thaliana* mesophyll protoplasts and observed by fluorescence microscopy. The merged pictures include the green fluorescence channel (first panels) and the chloroplast autofluorescence channel (second panels). The corresponding bright field images are shown on the right. Bars = 10 μm.

The cellular localization of Hsfs is essential to their functions (Scharf et al., 1998). To investigate the subcellular localization of FvHsfs, the coding-sequences of 14 *FvHsf* genes were fused in-frame with GFP under control of the CaMV *35S* promoter. The fusion constructs and the positive control were transiently expressed in *Arabidopsis* mesophyll protoplasts. As shown in Figure 4, the GFP expressed from the control construct was dispersed throughout the whole cell, whereas the most of the FvHsf fusion proteins were clearly located in the nucleus. However, some of the FvHsf fusion proteins, including FvHsfA2a-GFP, FvHsfA3a-GFP, FvHsfA4a-GFP, FvHsfA5a-GFP, FvHsfB2b-GFP, and FvHsfC1a-GFP, were also detected in the cytosol. To confirm the nuclear and cytosolic localization of the FvHsfs, we also examined the subcellular localization of VpCDPK2, a grape calcium-dependent protein kinase that was reported to localize to both the nucleus and cytosol of *Arabidopsis* mesophyll protoplasts (Zhang et al., 2015). The FvHsfs and VpCDPK2 exhibited similar subcellular distributions (Figure 4). These results might have broader implications for understanding the subcellular localization of plant Hsfs (Lyck et al., 1997; Kotak et al., 2004).

### Expression profiles of *FvHsfs* in different organs of the strawberry plant

**Figure 5:**
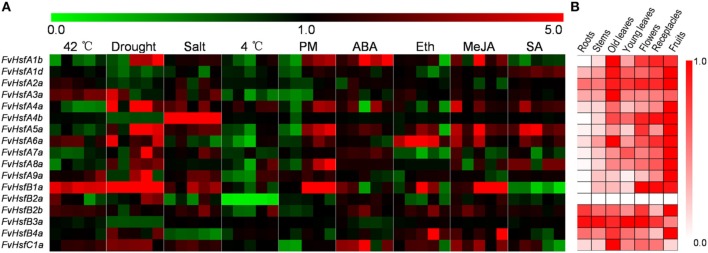
**The expression pattern of 17 ***FvHsf*** genes in different organs/tissues and under different treatments**. The expression profiles were generated by semi-quantitative PCR and were visualized as heat maps. The color scale represents log2 expression values, with red denoting increased transcript abundance and green denoting decreased transcript abundance. Only partial data are shown for the stress and hormone treatments (the expression levels of 17 *FvHsfs* at 0.5 hpt, 2 hpt, 4 hpt, 24 hpt, and 48 hpt of heat, cold, salt, and hormone treatments; the expression levels of 17 *FvHsfs* at 24 hpt/i, 48 hpt/i, 72 hpt/i, 144 hpt/i, and 168 hpt/i of drought treatment and powdery mildew infection). Detailed expression profiles of all 17 *FvHsfs* in response to all treatments are provided in Supplementary Figures S1, S2. **(A)** The expression profiles of the 17 *FvHsfs* in response to different treatments. **(B)** The organ/tissue distribution of 17 *FvHsf* genes under homeostatic conditions, with the red marks indicating the relative level of each *FvHsf* transcript in different organs/tissues.

To determine the biological roles of *Hsf* genes in strawberry Heilongjiang-3, the distribution of 17 *FvHsfs* transcripts was surveyed in six major organs (roots, stems, leaves, flowers, receptacles, and fruits) under non-stress conditions. As shown in Figure 5B, the *FvHsfA1d, FvHsfA2a*, and *FvHsfB3a* transcripts showed consistent distribution throughout all of the tested organs, and most of the *FvHsf* genes exhibited high mRNA levels in the fruits of Heilongjiang-3, with the exception of *FvHsfB2a* and *FvHsfC1a*. The *FvHsfA1b, FvHsfA4b, FvHsfA5a, FvHsfB1a, FvHsfB2b*, and *FvHsfC1a* genes showed high transcription levels in the flowers and receptacles of Heilongjiang-3; *FvHsfA1b, FvHsfA3a, FvHsfA4a, FvHsfA6a*, and *FvHsfC1a* also exhibited higher transcript abundance in old leaves than in roots, stems, and young leaves. In addition, the *FvHsfA1b, FvHsfA4b, FvHsfA5a, FvHsfA8a, FvHsfA9a*, and *FvHsfB1a* transcripts could not be detected in the roots of Heilongjiang-3, while *FvHsfB2a* could not be detected in any Heilongjiang-3 tissues. The organ-specific *FvHsf* expression patterns suggest that FvHsfs could play important biological roles in the growth and development of strawberries (Kotak et al., 2007).

### *FvHsfs* act as positive responders to heat stress in strawberry

**Figure 6:**
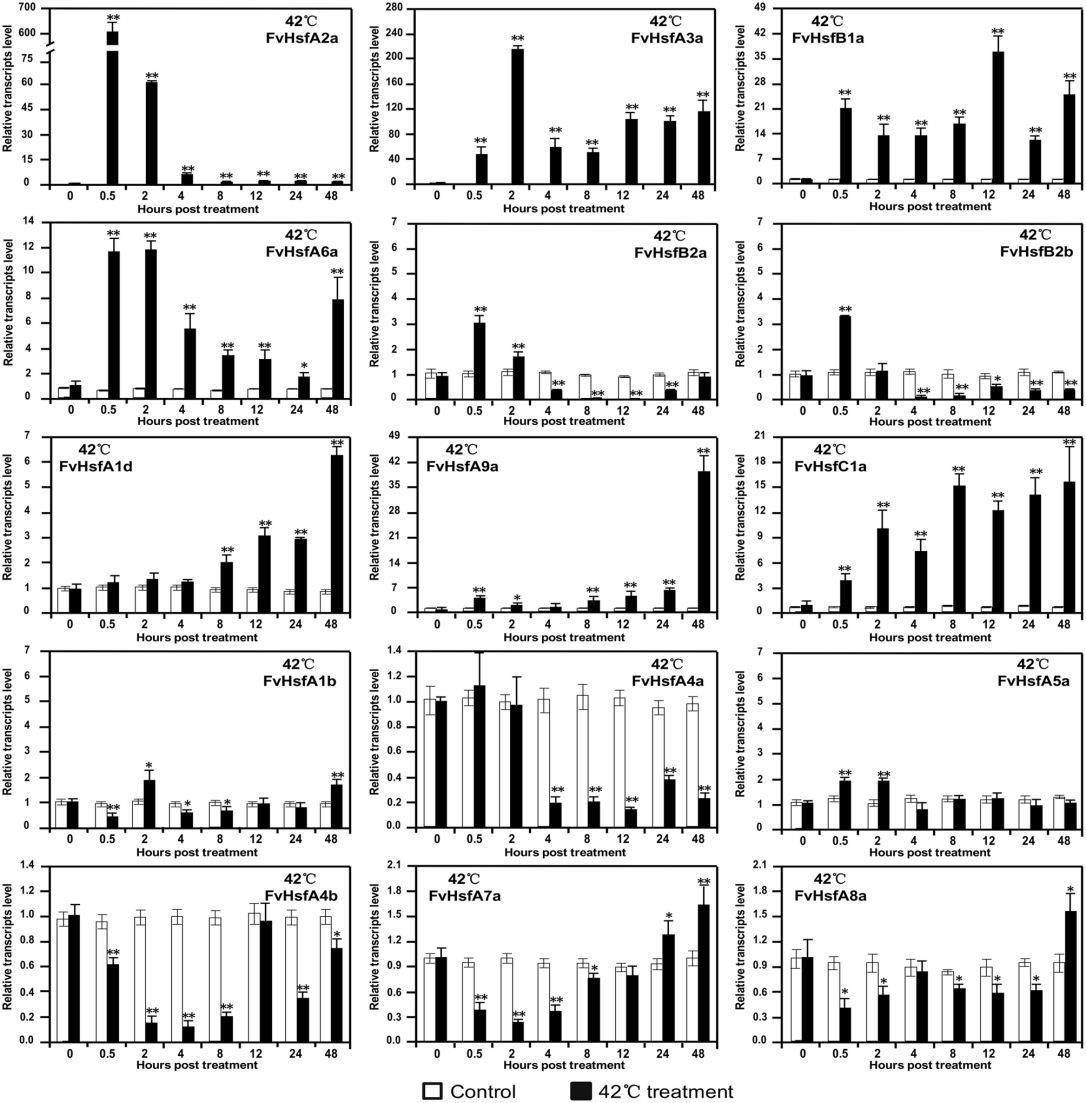
**The expression of ***FvHsf*** genes in the diploid woodland strawberry (***F. vesca***) in response to 42°C treatment**. Real-time quantitative PCR was performed to determine the expression of various *FvHsfs* in 42°C temperatures relative to control temperatures. The analysis results were normalized using *Fv18s*. The experiments were repeated three times and gave consistent results. The mean values and SDs were obtained from three biological and three technical replicates. The asterisks indicate that the corresponding gene was significantly up or down-regulated in response to treatment, as determined by the Student's *t*-test (^*^P < 0.05, ^**^P < 0.01).

To understand how *Hsf* genes respond to heat stress in strawberry, we exposed Heilongjiang-3 to 42°C conditions and performed semi-quantitative RT-PCR to determine expression profiles for the *FvHsf* gene family (Figure 5A and Supplementary Figure S1). Based on the results of the semi-quantitative RT-PCR, we selected 15 characteristic *FvHsfs* using real-time quantitative RT-PCR to further test their transcript abundance during 42°C treatment (Figure 6).

As shown in Figure 6, the 15 *FvHsf* genes showed distinct expression patterns during 42°C treatment. Of the 15 *FvHsfs, FvHsfA2a* was the most strongly heat stress induced *Hsf* gene in strawberry, reaching its highest transcription level (~600-fold) at 0.5 hpt. *FvHsfA3a, FvHsfA6a*, and *FvHsfB1a* were also strongly induced at 0.5 hpt, maintaining elevated mRNA levels (~10- to 200-fold) throughout the entire treatment period. *FvHsfA1d, FvHsfA9a*, and *FvHsfC1a* were highly induced at the late stage (24–48 hpt) of 42°C treatment, with these transcripts up-regulated 6- to 40-fold during this time period. *FvHsfA5a, FvHsfB2a*, and *FvHsfB2b* were up-regulated in the earlier stage of treatment (0.5–2 hpt), but returned to baseline or were down-regulated after 2 hpt. *FvHsfA4a, FvHsfA4b, FvHsfA7a*, and *FvHsfA8a* were primarily down-regulated in response to treatment, but *FvHsfA7a* and *FvHsfA8a* returned to baseline at the late stage of treatment. *FvHsfA1b* was only slightly up-regulated at 2 and 48 hpt.

### *FvHsfs* are associated with other abiotic and biotic stresses in strawberry

**Figure 7:**
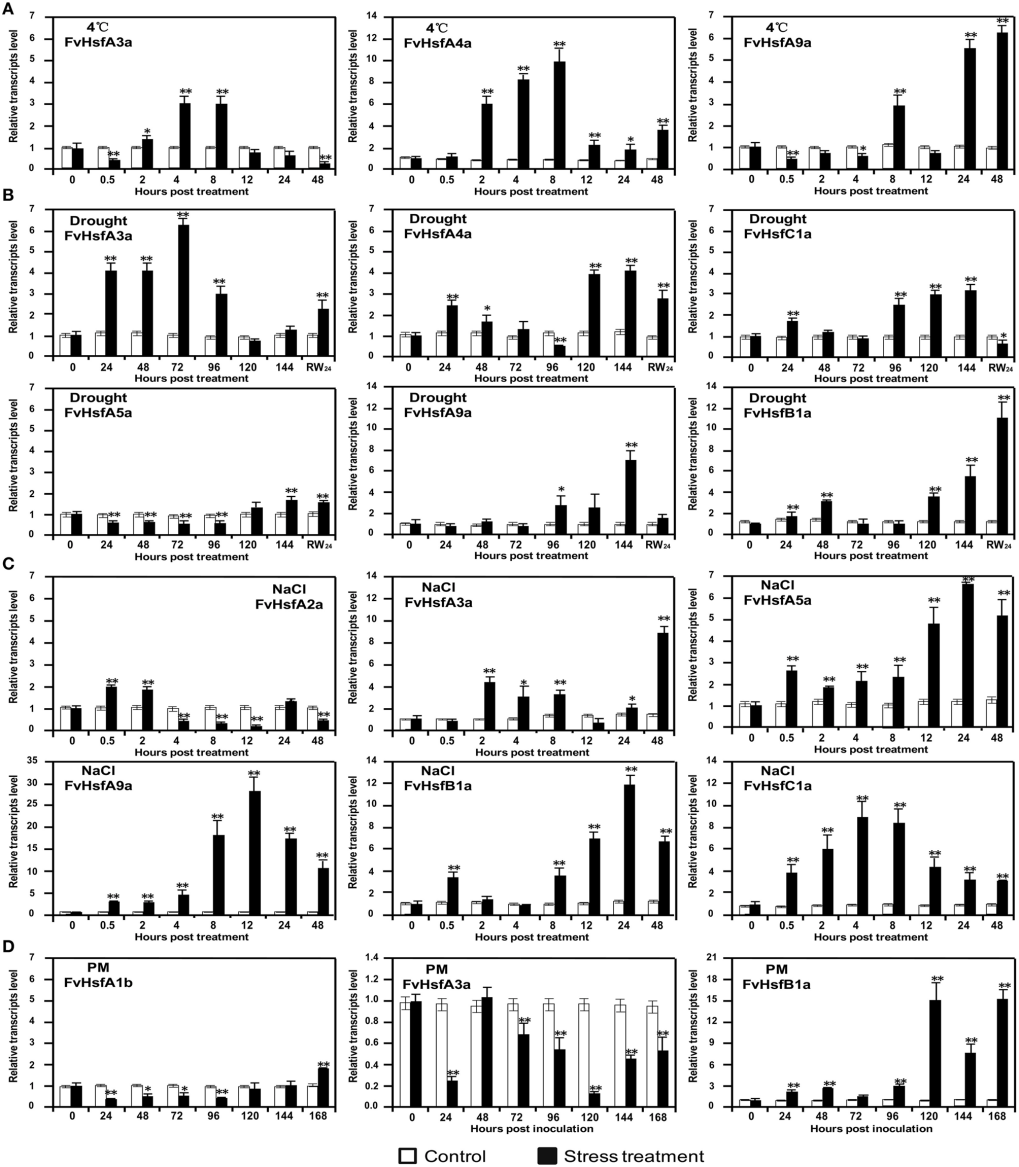
**The expression of ***Hsf*** genes in the diploid woodland strawberry (***F. vesca***) in response to 4°C, drought, salt, and ***Podosphaera aphanis*** (PM) treatments**. The expression levels of several representative *FvHsf* genes showed unusual patterns in response to 4°C **(A)**, drought **(B)**, salt **(C)**, and PM **(D)** treatments.

**Figure 8:**
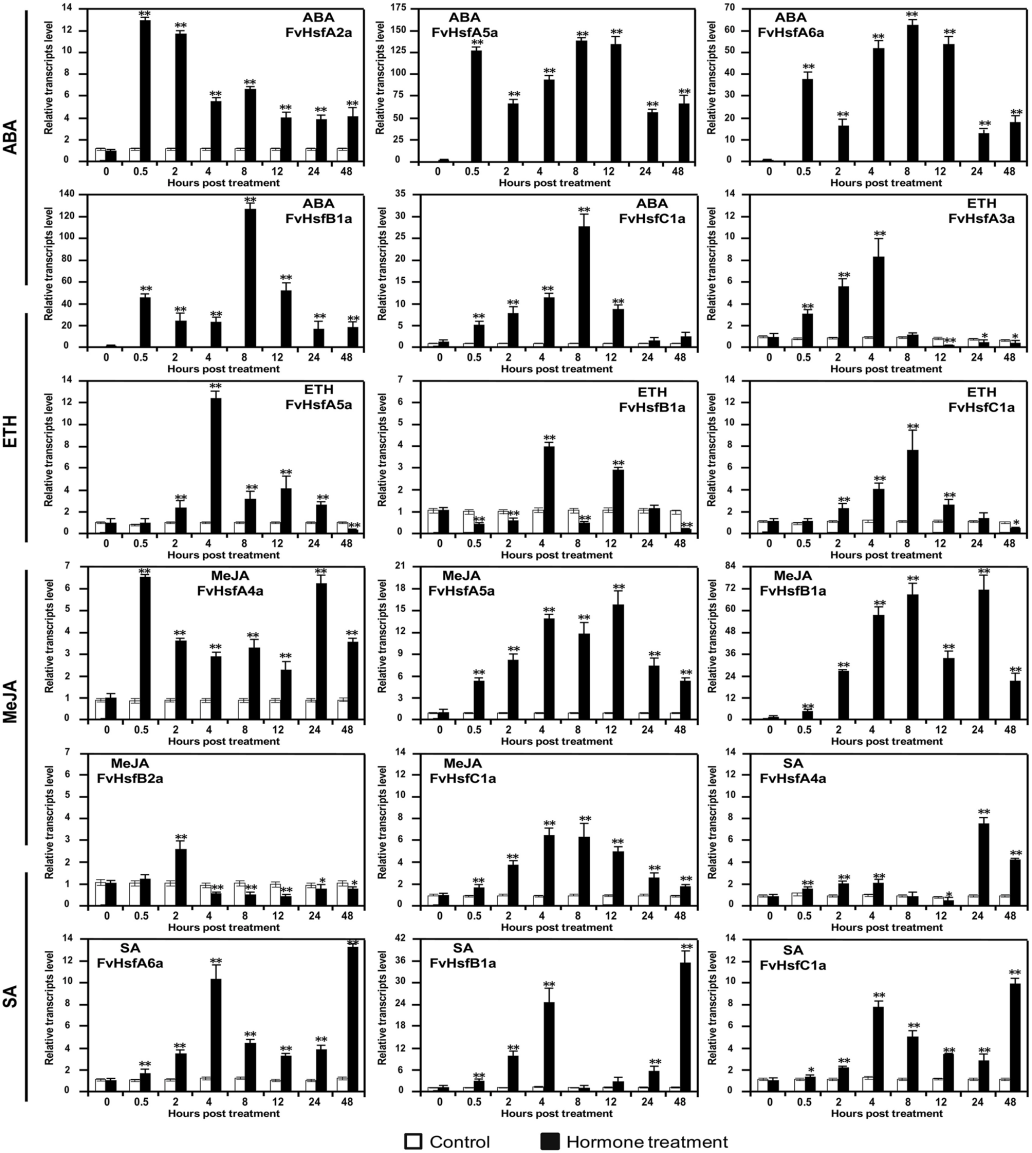
**The expression of ***Hsf*** genes in the diploid woodland strawberry (***F. vesca***) in response to treatment with plant hormones**. The expression levels of several representative *FvHsf* genes showed unusual patterns in response to ABA, SA, MeJA, and ethylene (Eth) treatments.

Although *Hsfs* are primarily involved in heat acclimatization, these genes have also been reported to play roles in plant adaptations to other environmental stresses, including cold (Mittal et al., 2009), salt (Hwang et al., 2014), drought (Prieto-Dapena et al., 2008; Hwang et al., 2014) and pathogen infection (Pick et al., 2012). In this study, the semi-quantitative RT-PCR indicated that most of the *FvHsfs* (*FvHsfA1b, A2a, A3a, A4a, A5a, A6a, A8a, A9a, B1a, B2a, B2b, B4a*, and *C1a*) showed detectable changes at the transcript level when strawberry plants were exposed to specific stress conditions (Figure 5A; Supplementary Figures S1, S2). These data suggest that these *FvHsfs* are involved in the response to almost all stress treatments, but examination with real-time quantitative RT-PCR revealed that the expression of each *FvHsf* is highly specific (Figures 7, 8).

*FvHsfA3a* and *FvHsfA4a* were up-regulated 3- and 10-fold, respectively, during the 2–8 hpt period after 4°C treatment, whereas *FvHsfA9a* was up-regulated over 3-fold during the 24–48 hpt period of the same treatment (Figure 7A). *FvHsfA3a* was highly up-regulated 24–96 hpt after drought treatment, whereas *FvHsfA4a, FvHsfB1a*, and *FvHsfC1a* were initially up-regulated within 48 hpt and *FvHsfA4a, FvHsfA5a, FvHsfA9a, FvHsfB1a*, and *FvHsfC1a* were highly induced 96 hpt (Figure 7B). The *FvHsfA4a, FvHsfA5a, FvHsfA9a*, and *FvHsfC1a* transcripts returned to baseline or decreased when the plants were rewatered, but *FvHsfB1a* remained up-regulated 11-fold at this time point (Figure 7B). In addition, the *FvHsfA2a* transcript was up-regulated in the earlier stage of salt stress treatment before decreasing to its lowest level at 12 hpt (Figure 5A; Supplementary Figure S1). In contrast, the *FvHsfA3a, FvHsfA5a, FvHsfA9a*, and *FvHsfB1a* transcripts were primarily up-regulated at the middle or late stage of salt treatment. The level of the *FvHsfC1a* transcript first increased and then decreased during salt treatment (Figure 7C). After inoculation with *Podosphaera aphanis, FvHsfB1a* levels gradually increased during the earlier stage (24–96 hpi) and dramatically increased 120 hpi, whereas *FvHsfA1b* and *FvHsfA3a* were clearly down-regulated during the earlier stage (Figure 7D).

### *FvHsf* expression in response to hormone treatments

Phytohormones such as abscisic acid (ABA), ethylene (Eth), salicylic acid (SA), and jasmonic acid (MeJA) have well-established roles in plant stress signaling networks (Kotak et al., 2007a; Clarke et al., 2009). To assess the potential roles of *FvHsfs* in phytohormone-mediated signal transduction, we examined the transcriptional abundance of 17 *FvHsfs* during ABA, Eth (ethephon), SA, and MeJA (Methyl jasmonate) treatments. Quantitative RT-PCR analysis revealed that ABA treatment causes the rapid (0.5 hpt) up-regulation of several *FvHsf* genes, including *FvHsfA2a, FvHsfA5a, FvHsfA6a, FvHsfB1a*, and *FvHsfC1a*. The levels of these transcripts remained higher (4- to 125-fold) throughout the entire treatment period (Figure 8). During Eth treatment, *FvHsfA3a, FvHsfA5a*, and *FvHsfC1a* were gradually up-regulated by 7.5- to 12-fold within 8 hpt, whereas *FvHsfB1a* was only up-regulated at 4 and 12 hpt. Treatment with MeJA also increased *FvHsfA4a, FvHsfA5a, FvHsfB1a*, and *FvHsfC1a* levels significantly (6- to 70-fold) throughout the entire treatment period, while *FvHsfB2a* was only up-regulated at 2 hpt before returning to a lower (~0.5-fold) abundance. During SA treatment, the *FvHsfA4a, FvHsfA6a, FvHsfB1a*, and *FvHsfC1a* transcripts exhibited a continuous increase: the transcript levels first peaked at 4 hpt and were again up-regulated during the 24–48 hpt time period (Figure 8). It is worth noting that *FvHsfA4a, FvHsfA5a, FvHsfA6a, FvHsfB1a*, and *FvHsfC1a* were clearly involved in the response to all four of the hormone treatments (Figures 5A, 8).

## Discussion

*F. vesca*, a diploid woodland strawberry with a small and sequenced genome, is an excellent model for studying the counterparts of many important genes in the Rosaceae fruit crops (Shulaev et al., 2011; Darwish et al., 2015). An increasing amount of evidence has indicated that *Hsf* genes play essential roles in plant adaptations to various stress conditions (Shen et al., 2015; Xue et al., 2015). To explore Hsf functions in abiotic and biotic stress responses in the strawberry, this study identified and isolated *Hsf* genes in a diploid woodland strawberry in addition to analyzing the evolutionary relationships, gene structure, protein domains, subcellular localization, and expression profiles of these genes in response to abiotic or biotic stresses and hormone treatments.

### The *Hsf* gene family is conserved in strawberry

The size of the strawberry *Hsf* gene family (17) is smaller than that of other plant species, such as *Arabidopsis* (21) (Scharf et al., 2012), rice (25) (Guo et al., 2008), apple (25) (Giorno et al., 2012), wheat (56) (Xue et al., 2014), and soybean (59) (Chung et al., 2013). The small size of the strawberry genome (Shulaev et al., 2011; Darwish et al., 2015) and lack of the large genome duplications seen in apple (Velasco et al., 2010), soybean (Schmutz et al., 2010), and wheat (Mayer et al., 2014) could explain the limited size of the *FvHsf* family (Figure 1A).

The comparative genomics approach structures genomes into syntenic blocks that exhibit conserved features (Ghiurcuta and Moret, 2014). This synteny analysis provided evolutionary and functional connections between genes in strawberry and *Arabidopsis*. Eleven strawberry *Hsf* genes were found to have syntenic relationships with *Arabidopsis* genes (Figure 1B), suggesting that most of the strawberry *Hsf* genes might have arisen before the divergence of the *Arabidopsis* and strawberry lineages. All of these *FvHsf* genes show close phylogenetic relationships with the corresponding *AtHsf* genes (Figure 1A), suggesting the potential for functional similarities. Structural characteristics such as intron-exon structures and protein domains might reflect the functional conservation or differentiation of the gene family. Fourteen of the seventeen *FvHsf* genes have only one phase zero intron while the others lack this intron (Figure 2), suggesting that the *FvHsf* gene family is conserved (de Souza et al., 1998). Besides, all 17 of the FvHsf proteins contain the necessary and/or specific protein domains (DBD, OD, NLS, NES, and AHA), which might be essential for functional conservation (Giorno et al., 2012). Fourteen of the FvHsf proteins retained the ability to localize in the nucleus, suggesting that most (14/17) of the *FvHsf* genes still maintain ancestral functional features (Figure 4).

### *FvHsf* genes could play essential roles in strawberry adaptation to abiotic or biotic stresses

The function of plant Hsfs is to elicit the expression of genes encoding heat shock proteins (Hsps) or other stress-inducible genes (Schramm et al., 2006; Chan-Schaminet et al., 2009; Nishizawa-Yokoi et al., 2009) that are known to play a central role in protecting plants from heat or other stress conditions (Scharf et al., 2012). Recent genome-wide expression profile analyses of crop plants (rice, wheat, soybean, and apple) indicated that several *Hsf* genes are transcribed at high levels during heat, cold, salt, and drought stresses (Mittal et al., 2009; Giorno et al., 2012; Chung et al., 2013). In this study, 15 *FvHsf* genes showed distinct expression patterns during heat treatment (Figure 6), with most of these genes also were induced by other abiotic or biotic stresses and hormone stimuli (Figures 7, 8). It is notable that the subcellular localization of each FvHsf protein was different (Figure 4). All of our data suggest that the *FvHsfs* are involved in the response to almost all of the stress treatments, but these genes are highly specific in function.

*FvHsfA2a*, whose rice orthologs are *OsHsfA2a/b/e*, was found in the chromosomal region syntenic with *AtHsfA2a* (Figure 1). The characteristics of *FvHsfA2a* are highly consistent with the characteristics of *OsHsfA2* and *AtHsfA2a*. For example, *OsHsfA2a* and *AtHsfA2a* have been reported as the most strongly induced *Hsfs*, accumulating to high levels in plants exposed to long-term heat stress (Schramm et al., 2006; Mittal et al., 2009). This finding is consistent with our results showing that *FvHsfA2a* was the most strongly induced of the 17 *FvHsfs* (~600-fold) in response to heat stress (Figure 6). In addition, *AtHsfA2a* was reported to play a broader role in the expression of multiple Hsps (Hsp70-5, Hsp18.1-Cl, and Hsp22-ER) (Schramm et al., 2006) and general stress-related non-chaperone encoding genes such as GOLS1 or APX2 (Nishizawa-Yokoi et al., 2009), while the *FvHsfA2a* transcript was clearly up-regulated in response to salt and ABA treatments (Figures 7C, 8). Lastly, while SlHsfA2a, a homolog of FvHsfA2a in tomato, reportedly localizes to the nucleus, interaction with SlHsfA1 is required for the efficient nuclear import of SlHsfA2a (Scharf et al., 1998). This finding is consistent with our results showing that FvHsfA2a-GFP fusion protein also localized to nucleus but could also be detected in the cytosol of *Arabidopsis* mesophyll protoplasts, which may result from the absence of the specific interaction factor (Figure 4).

*FvHsfA3a* has orthologs in apple (*MdHsfA3a/b/c*) and has a syntenic gene in *Arabidopsis* (*AtHsfA3a)* (Figure 1). *MdHsfA3b/c* are involved in maintaining the stress response when apple trees were exposed to prolonged periods of high temperature (Giorno et al., 2012). *AtHsfA3a* expression was regarded as part of the drought stress response (Schramm et al., 2008). *SlHsfA3a*, a homolog of *FvHsfA3a* in tomato, is a nuclear-localized Hsf that is induced by heat stress, high salinity, and drought, but not abscisic acid (Li et al., 2013). Our data are highly similar, with *FvHsfA3a* accumulating during heat, drought, salt, and ethephon treatments (Figures 6, 7B,C, 8) and FvHsfA3a-GFP fusion proteins clearly localized to the nucleus of protoplasts (Figure 4).

*FvHsfA4a* and *FvHsfA5a* are intriguing strawberry *Hsfs*. *FvHsfA4a* and *FvHsfA5a* were the earliest *FvHsfs* to diverge from the evolutionary branch, with similar results being found in the Hsf families of other species (Figure 1A). In addition, both FvHsfA4a and FvHsfA5a show significant localization to the cytosol of *Arabidopsis* mesophyll protoplasts (Figure 4). Notably, *FvHsfA4a* and *FvHsfA5a* were both distinctly up-regulated in response to abiotic stress (cold, drought, and salt) (Figure 7) and hormone treatments (ABA, Eth, MeJA, and SA) (Figure 8). The translocation of Hsf proteins from the cytosol to the nucleus is redox-dependent (Giesguth et al., 2015), and *AtHsfA4a*, an *Arabidopsis* ortholog of *FvHsfA4a*, has been shown to play a central role in the early sensing of H_2_O_2_ stress in *Arabidopsis* (Davletova et al., 2005). Thus, we speculate that the dramatically increased expression of *FvHsfA4a* and *FvHsfA5a* in response to a range of treatments (Figures 6–8) and the specific cellular localization of FvHsfA4a and FvHsfA5a (Figure 4) may have important implications for stress signaling.

It is notable that *FvHsfA6a* is an important member of the *FvHsf* family, with two syntenic *AtHsfs* in *Arabidopsis*: *AtHsfA6b* and *AtHsfA7b* (Figure 1B). Yoshida et al. confirmed that *AtHsfA6a/b* were involved in ABA-dependent signaling in response to water deficiency stress (Yoshida et al., 2010), and our data also showed that *FvHsfA6a* was highly expressed in response to ABA treatment (Figure 8). Recently, Xue et al. demonstrated that *TaHsfA6f*, a *FvHsfA6a* ortholog in *Triticum aestivum*, serves as a transcriptional activator that regulates a suite of heat stress protection genes in wheat (Xue et al., 2015). This finding is consistent with *FvHsfA6a* showing high expression under heat treatment and highly specific nuclear localization (Figures 4, 6).

Undoubtedly, *FvHsfA9a* is a unique member of the *FvHsf* family. While *FvHsfA9a* is phylogenetically close to *FvHsfA2a* (Figure 1A), the expression characteristics of *FvHsfA9a* are entirely different from *FvHsfA2a*, which was primarily induced at the earlier stage of treatments (Figures 6, 7C, 8). In contrast, the current data identified *FvHsfA9a* as a late response factor in long-term heat, drought, salt, and cold stress treatments (Figures 6, 7). The *Arabidopsis* and *Helianthus annuus* homologs of *FvHsfA9a* function as seed-specific sHsp (small heat shock protein) regulators (Kotak et al., 2007; Prieto-Dapena et al., 2008) and are associated with ABA-mediated stress signaling and drought resistance (Schramm et al., 2008). Similarly, *FvHsfA9a* showed specific nuclear localization (Figure 4) and was only induced in response to ABA (Figure S2).

It is worth noting that *FvHsfB1a* was induced in response to all of the stress or hormone treatments, and was both instantly induced by all stimuli and also reached a high expression level during all of the treatments (Figures 6–8). Bharti et al. provided evidence that *SlHsfB1*, the tomato ortholog of *FvHsfB1a*, represents a novel type of coactivator, cooperating with class A Hsfs or other activators that control housekeeping gene expression during stress conditions (Bharti et al., 2004). Moreover, *FvHsfB1a* was highly induced at the late stage of powdery mildew infection (Figure 7) and accumulated to high levels during SA and MeJA treatments (Figure 8). Similarly, *AtHsfB1a*, the *Arabidopsis* homolog of *FvHsfB1a*, was reported as a crucial component of salicylic acid-mediated resistance (Kumar et al., 2009; Ikeda et al., 2011; Pick et al., 2012), and subsequent evidence also demonstrated that *AtHsfB1a* plays a pivotal role in primed defense gene activation and the pathogen-induced acquired immune response (Pick et al., 2012). Therefore, it is reasonable to speculate that *FvHsfB1a* is a key player in the acquired immune response to the biotrophic fungus *Podosphaera aphanis*.

In addition to stress, *Hsfs* have also been reported to play roles in plant growth and development (Begum et al., 2013). The detailed expression profiles of individual *FvHsfs* in six important organs were determined in this study (Figure 5B). Notably, several *FvHsfs* (*FvHsfA2a, A3a, A4a, A5a, A6a, A9a, B1a*, and *C1a*) exhibited organ-specific distribution and were clearly involved in stress responses (Figure 5A), suggesting that FvHsf members could be important in protecting growing or developing strawberry plants from heat damage.

## Conclusion

In this study, we identified 17 *FvHsf* genes in a diploid strawberry by employing bioinformatics and publicly available data. The evolutionary relationship and structural feature analyses have aided in identifying the potential functions of individual strawberry *Hsfs* by retrieving clues from the well-investigated *Arabidopsis*. Numerous *cis*-acting elements were found in the *FvHsf* promoter sequences, suggesting that *FvHsf* gene expression is controlled by a complex regulatory regime. The gene expression profiles obtained during 42°C, 4°C, drought, salt, powdery mildew infection, and phytohormone treatments suggest that several strawberry *Hsf* genes (including *FvHsfA2a, FvHsfA3a, FvHsfA4a, FvHsfA5a, FvHsfA6a, FvHsfA9a, FvHsfB1a*, and *FvHsfC1a*) could play important roles in adaptation to environmental stresses. In addition, the distinct subcellular localization of strawberry Hsfs suggests the potential functional divergence of several strawberry Hsfs. Taken together, the present work may provide the basis for further studies to dissect *FvHsf* function in response to stress stimuli.

## Author contributions

JF conceived and designed the experiments. YH, WW, and YTH performed the experiments. YH, KZ, WW, and JF analyzed the data. YH, YL, YG, and FZ contributed reagents/materials/analysis tools. YH and JF contributed to the writing of the manuscript. The authors thank Dr. Ke Duan of the Shanghai Academy of Agricultural Sciences for generously providing wild type *Fragaria vesca* plants.

### Conflict of interest statement

The authors declare that the research was conducted in the absence of any commercial or financial relationships that could be construed as a potential conflict of interest.

## Supplementary material

Supplementary Figure S1 **Expression profiles of 17 *FvHsf* genes in response to abiotic and biotic stress treatments, as determined using semi quantitative RT-PCR**.

Supplementary Figure S2 **Expression profiles of 17 *FvHsf* genes in response to phytohormone treatments, as determined using semi quantitative RT-PCR**.

Supplementary Table S1 **The primers used in this study**.

Supplementary Table S2 **The best matches for the protein motif sequences**.

Supplementary Table S3 **The syntenic relationships between strawberry and *Arabidopsis Hsf* genes**.
