# Supplementary figures and images for: Identification, isolation, and expression analysis of heat shock transcription factors in the diploid woodland strawberry Fragaria vesca
Source: Front Plant Sci. 2015 Sep 15;6:736. doi: 10.3389/fpls.2015.00736 (PMC4569975; doi:10.3389/fpls.2015.00736)

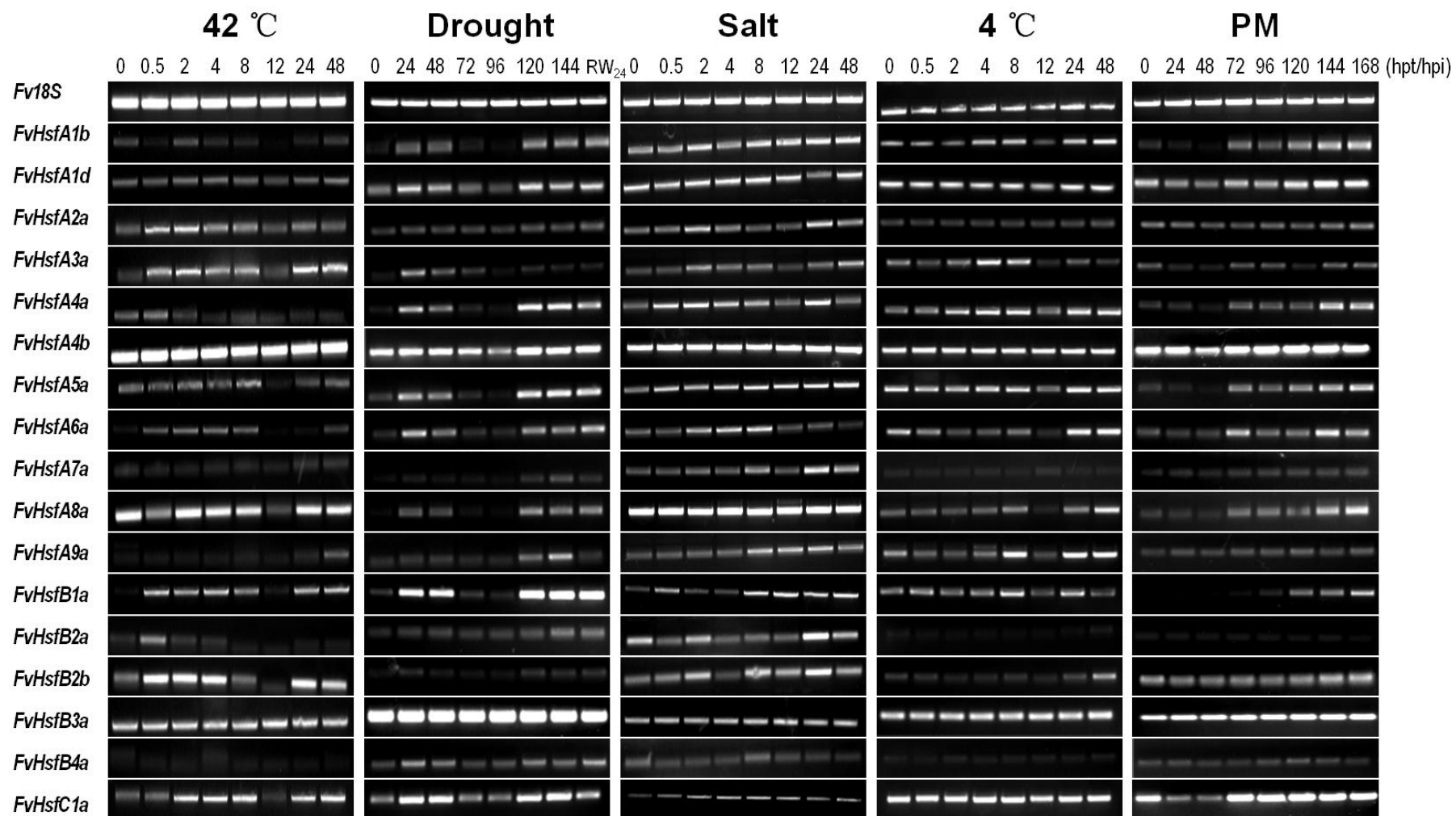

Supplement: Supplementary Figure S1 — Expression profiles of 17 FvHsf genes in response to abiotic and biotic stress treatments, as determined using semi quantitative RT-PCR. [file Image1.PDF]

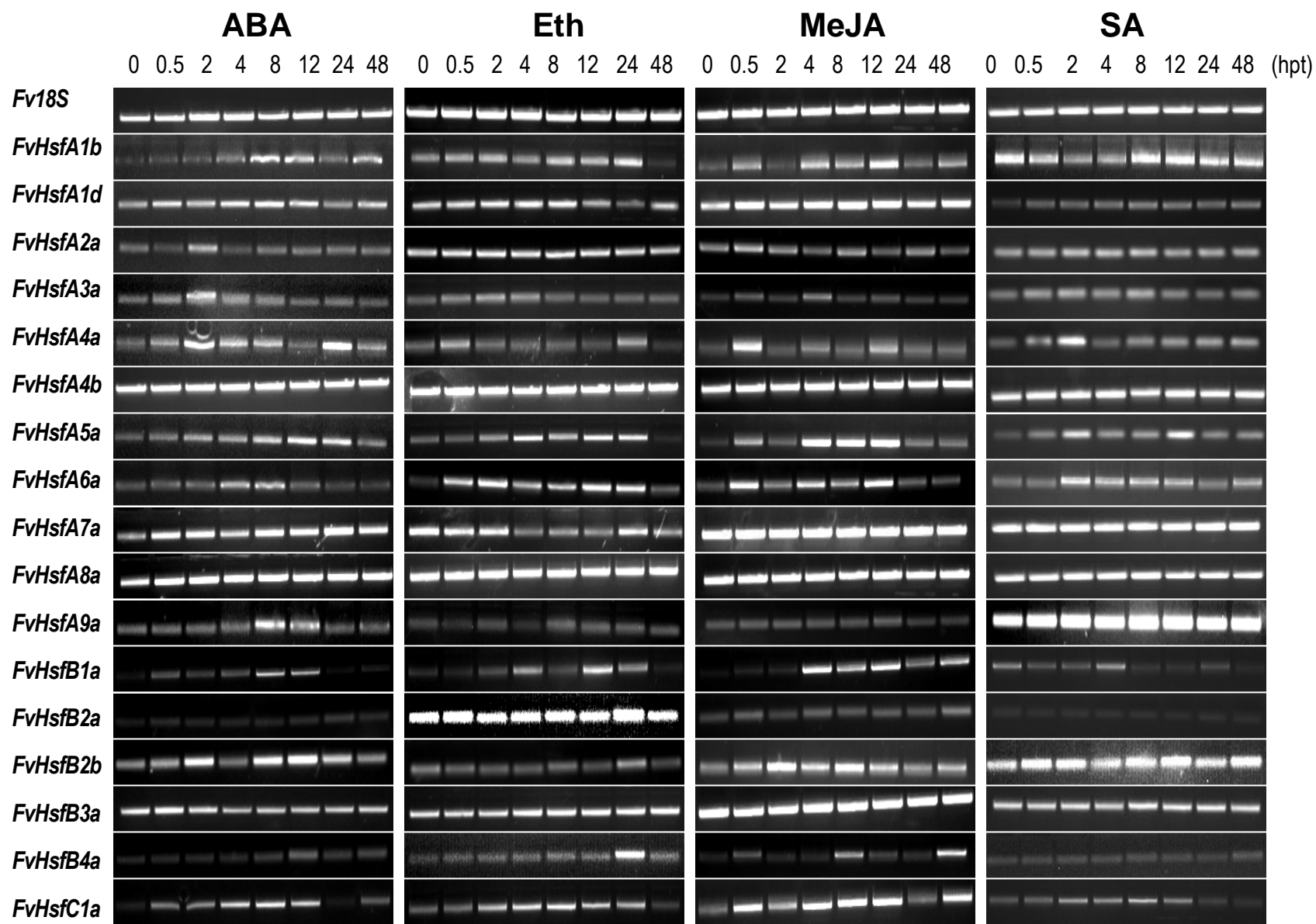

Supplement: Supplementary Figure S2 — Expression profiles of 17 FvHsf genes in response to phytohormone treatments, as determined using semi quantitative RT-PCR. [file Image2.PDF]
