# Supplementary material for: Identification, isolation, and expression analysis of heat shock transcription factors in the diploid woodland strawberry Fragaria vesca
Source: Front Plant Sci. 2015 Sep 15;6:736. doi: 10.3389/fpls.2015.00736 (PMC4569975; doi:10.3389/fpls.2015.00736)
Supplement: Supplementary Table S1 — The primers used in this study. [file Table1.PDF]

Supplementary Table S1. The primers used in this study.

Primer Names	Sequence (5'-3')[1]	Description[2]
FvHsfA1b-F	GAGAACACGGGGGACTCTAGAATGGAGGGAATCGAAGACACG	cloning
FvHsfA1b-R	TTCTCCTTTACCCATGGTACCACCAATCATGCCCTCAAGCC	cloning
FvHsfA1d-F	GAGAACACGGGGGACTCTAGAATGGTCGGTACTAGTACCAACCCCGAC	cloning
FvHsfA1d-R	ACCCATGGTACCCCGCTCGAGAACCCCTTTAGTATTTGATTTTAG	cloning
FvHsfA2a-F	GAGAACACGGGGGACTCTAGAATGGAGACAGTGATAGTGAAAGAA	cloning
FvHsfA2a-R	TTCTCCTTTACCCATGGTACCAGGATTGGCCTTGATCAGATA	cloning
FvHsfA3a-F	GAGAACACGGGGGACTCTAGAATGTTATCTGGTTTTCGCAAG	cloning
FvHsfA3a-R	TTCTCCTTTACCCATGGTACCTCCATCTTGACTTGCACGC	cloning
FvHsfA4a-F	GAGAACACGGGGGACTCTAGAATGGATGAAGTTCAAGGCATT	cloning
FvHsfA4a-R	ACCCATGGTACCCCGCTCGAGTGTGTTCTCTACTGAACTAAGATG	cloning
FvHsfA5a-F	GAGAACACGGGGGACTCTAGAATGGACGGAACCGCGGG	cloning
FvHsfA5a-R	ACCCATGGTACCCCGCTCGAGAAGAGTAAGTGTTTCTGTACCTTTAGC	cloning
FvHsfA6a-F	GAGAACACGGGGGACTCTAGAATGAATTACTTGTATCCAGTGAAGG	cloning
FvHsfA6a-R	TTCTCCTTTACCCATGGTACCACTTGGGGTTGAACCTAAGTA	cloning
FvHsfA9a-F	GAGAACACGGGGGACTCTAGAATGGTCGTTTCTGTTGGTGGT	cloning
FvHsfA9a-R	TTCTCCTTTACCCATGGTACCAGGCATTGATCCAACACATCC	cloning
FvHsfB1a-F	GAGAACACGGGGGACTCTAGAATGGCGCAAAGGTCAGTTCCG	cloning
FvHsfB1a-R	TTCTCCTTTACCCATGGTACCGTTGCACACCTTCCCGCTCTT	cloning
FvHsfB2a-F	GAGAACACGGGGGACTCTAGAATGGCTCCTCTGCCGGTGGAG	cloning
FvHsfB2a-R	ACCCATGGTACCCCGCTCGAGATTACACACCCTCTGATTGGC	cloning
FvHsfB2b-F	GAGAACACGGGGGACTCTAGAATGGCTTCGTTGCCGGACCAG	cloning
FvHsfB2b-R	ACCCATGGTACCCCGCTCGAGCTTCCGTAGCTCCAGCC	cloning
FvHsfB3a-F	GAGAACACGGGGGACTCTAGAATGGAGGGTGTGTGTGATCAA	cloning
FvHsfB3a-R	ACCCATGGTACCCCGCTCGAGTTTGCATGGTTGAGATAGTAA	cloning
FvHsfB4a-F	GAGAACACGGGGGACTCTAGAATGGCTTTGATGATAGACAACTG	cloning
FvHsfB4a-R	TTCTCCTTTACCCATGGTACCACATGTGGATGGGGGCATAAGGT	cloning
FvHsfC1a-F	GAGAACACGGGGGACTCTAGAATGATGATAACTAACACCTCA	cloning
FvHsfC1a-R	TTCTCCTTTACCCATGGTACCAAACCCACCTTCTAACAG	cloning
FvHsfA1b-qF	TTTGAAGACTGTGAATAGGCGAAAGC	RT-qPCR
FvHsfA1b-qR	CCTGCACACGTTGACCAACATTTT	RT-qPCR
FvHsfA1d-qF	CCTGCACATGGACATAGTCTTCAACAG	RT-qPCR
FvHsfA1d-qR	GACGTTGGACCATTGTTTGCAGC	RT-qPCR
FvHsfA2a-qF	GAGGAGGAGGCATGTGTCGCAG	RT-qPCR
FvHsfA2a-qR	TTCTGTTGTTGCTGCCTCAATCTCAC	RT-qPCR
FvHsfA3a-qF	GCGCAAGTCATGTCAGTCCTTACAAAT	RT-qPCR
FvHsfA3a-qR	ATCTGCTTCTGTCTCTGCTCAGCAGAC	RT-qPCR
FvHsfA4a-qF	TAGACGAAAGCCGGTGCATAGTCATT	RT-qPCR
FvHsfA4a-qR	TGGATTGCTGCTGCTCTTGCATAT	RT-qPCR
FvHsfA4b-qF	GCGGCTTGAGAAAATGGAGTCTTC	RT-qPCR
FvHsfA4b-qR	AGACCCAGCCACCTCTGGGGAT	RT-qPCR

FvHsfA5a-qF	CAGCCACAGTAATCCCCAGGGTT	RT-qPCR
FvHsfA5a-qR	CTGCCTCTGCTCCATACCATTTACC	RT-qPCR
FvHsfA6a-qF	CTCATCAGCCTATTCCACCGCAC	RT-qPCR
FvHsfA6a-qR	CATGGCTTGAAGGCACGCTCTAG	RT-qPCR
FvHsfA7a-qF	GTTCATTATGCTAATTCACAGCAGGGT	RT-qPCR
FvHsfA7a-qR	CTCTTGAGCCTATCTTCCATTCCATG	RT-qPCR
FvHsfA8a-qF	TTCTCGGTTATGATGCTCCCCAAGT	RT-qPCR
FvHsfA8a-qR	CTCTGATCTGTGCCCTGCGGAT	RT-qPCR
FvHsfA9a-qF	GGGACTGCCATGAGTTCTCTAAGACC	RT-qPCR
FvHsfA9a-qR	ACACATTGCTTCCTTGTGGCTGC	RT-qPCR
FvHsfB1a-qF	GCTTCTCTCCGAGATTCGACGC	RT-qPCR
FvHsfB1a-qR	GCGTCTCGTTGTCTTTCTTCAGCTT	RT-qPCR
FvHsfB2a-qF	CATCTGCTCTGCGACATACAGCG	RT-qPCR
FvHsfB2a-qR	GACCTCCTTGGTCAGCCGGAGA	RT-qPCR
FvHsfB2b-qF	AATCTCTCCGTCGGTGTCCGCT	RT-qPCR
FvHsfB2b-qR	TATCTCCGCCGTGGAGTTGCAG	RT-qPCR
FvHsfB3a-qF	GTCGTAGAAAAGCATGGTCGAACAAG	RT-qPCR
FvHsfB3a-qR	CAAATCGAGAAGCTCCTTGCACTTG	RT-qPCR
FvHsfB4a-qF	ACGGCTCAGCCGACACAGCTC	RT-qPCR
FvHsfB4a-qR	AGTAGCCAGTGTTGAGGGGACCAC	RT-qPCR
FvHsfC1a-qF	GAGTTCTCGCAGCGGCTGTTG	RT-qPCR
FvHsfC1a-qR	GATTGCCCGTGTGTTTTCTCCTC	RT-qPCR
Fv18S-qF	ACCGTTGATTCGCACAATTGGTCATCG	RT-qPCR
Fv18S-qR	TACTGCGGGTCGGCAATCGGACG	RT-qPCR

1 Restriction sites are indicated in red.

2 The type of experiment for which the primers were used is indicated in brackets (C: cloning, QRT: RT-qPCR).
