# Supplementary material for: Identification, isolation, and expression analysis of heat shock transcription factors in the diploid woodland strawberry Fragaria vesca
Source: Front Plant Sci. 2015 Sep 15;6:736. doi: 10.3389/fpls.2015.00736 (PMC4569975; doi:10.3389/fpls.2015.00736)
Supplement: Supplementary Table S2 — The best matches for the protein motif sequences. [file Table2.PDF]

Supplementary Table S2. The best match for the sequences of protein motifs.

Motif Names	Sequence (5'-3')	Function
Motif 1, Motif 2, Motif 3	PPPFLSKTYDMVDDPATDHIISWSEAGNSFVVWDPHEFARDLLPKYFKHNNFSSFVRQLNTYGFRKIDPDRWEFANECFLRGQKHLLKNIHRRK	DBD
Motif 4	CVEVGKFGL	unknown
Motif 5	GLWDEIERLKRDK	OD
Motif 6	LKRDKNVLMQEIVRLRQQQQYTEHQMQAMNQRLQGMECRQQQMMSFLAKAMQNPGFL	OD (class A & C)
Motif 7	TWHLIEENERLRKENMMLSCELTQCKCKCNNIFYMIMNY	OD (class B)
Motif 8	QLVQHKNMKKRLIGGVNKKRRRPI	NLS
Motif 9	DGQIVKYQPPMNEAAKAM	unknown
Motif 10	FCRGSNSPEDSDDFPWWC	unknown
Motif 11	VNDVFWEQFLTE	AHA
Motif 12	WWNTRNVDNLTEQMGHL	NES
Motif 13	YHNDPKQTRPHHIPDPHIM	unknown
Motif 14	IGNGNFMAWQNVNRDYMDM	unknown
Motif 15	IIEKDHDLGMHLRPCSPC	unknown
Motif 16	YDGHDCM	unknown
Motif 17	MPEEVDDIGPM	unknown
Motif 18	EPARSVSL	unknown
Motif 19	NYLIEQAGC	unknown
Motif 20	PKLFGV	NLS
Motif 21	SGGGGG	unknown
Motif 22	YNKKRRL	NLS
Motif 23	PKPMEGLN	unknown
