# Supplementary material for: Identification, isolation, and expression analysis of heat shock transcription factors in the diploid woodland strawberry Fragaria vesca
Source: Front Plant Sci. 2015 Sep 15;6:736. doi: 10.3389/fpls.2015.00736 (PMC4569975; doi:10.3389/fpls.2015.00736)
Supplement: Supplementary Table S3 — The syntenic relationships between strawberry and Arabidopsis Hsf genes. [file Table3.PDF]

Supplementary Table S3. The syntenic relationships among strawberry and *Arabidopsis Hsf* genes.

Block	Arabidopsis gene location			Strawberry gene location			Arabidopsis gene ID	Strawberry gene ID	Strawberry gene name
	Chr	start	end	chr	start	end			
97	at1	11470056	11666058	LG5	15851861	16655550	AT1G32330	101302657	*FvHsfA1d*
207	at2	11041331	11150510	LG2	18122743	18457637	AT2G26150	101312795	*FvHsfA2a*
267	at2	17355331	17427282	LG6	15533362	16063062	AT2G41690	101307517	*FvHsfB3a*
333	at3	7969681	8154149	LG2	14278407	14634112	AT3G22830	101307359	*FvHsfA6a*
366	at3	8068516	8179743	LG4	393585	1176135	AT3G22830	101297339	*FvHsfA7a*
322	at3	8928169	8990873	LG1	1502666	1683467	AT3G24520	101304412	*FvHsfC1a*
332	at3	23353615	23422399	LG2	14300321	14608462	AT3G63350	101307359	*FvHsfA6a*
582	at4	7006648	7401907	LG5	1291203	195893	AT4G11660	101301205	*FvHsfB2b*
564	at4	7970200	8195265	LG4	16706934	17617941	AT4G13980	101302160	*FvHsfA5a*
581	at4	9856296	9880254	LG5	16304499	16675705	AT4G17750	101302657	*FvHsfA1d*
599	at4	17374458	17509747	LG6	35085957	35807354	AT4G36990	101294882	*FvHsfB1a*
758	at5	908563	1015345	LG6	12708790	11902522	AT5G03720	101295258	*FvHsfA3a*
784	at5	24916089	24979849	LG7	21835968	22184992	AT5G62020	101299242	*FvHsfB2a*
